# Chicago Medical Society, August 17th

**Published:** 1885-09

**Authors:** 


The society then adjourned.

---

### CHICAGO MEDICAL SOCIETY.

*Stated Meeting, Aug. 17, 1885.* THE PRESIDENT, PROFESSOR C. T. PARKES, M. D., in the chair.

### A CASE OF OVARIOTOMY

Was reported by PROFESSOR C. T. PARKES, as follows: In Feb., 1885, the patient, Mrs. R., aged forty-three, ten children, came under the care of Dr. O'Connell, of Ponca, Nebraska, who diagnosticated ovarian tumor. Professor Parkes was requested to perform ovariotomy. He found the patient in fair condition generally, hopeful and clear-minded. The abdomen was glistening with distension. Fluctuation was extremely well marked in all directions and in all positions of the patient. There was no resonance anywhere, and there was no umbilical hernia. Her trouble dated from August, 1884, when a small tumor was perceptible on the right side. It gradually increased in size until January 17, 1885, when local peritonitis set in and was followed by rapid distension. Ovariotomy was done

March 24, 1885. The patient had been placed in a bright light and an incision four inches in length made in the abdominal walls, midway between the umbilicus and pubes, to the peritoneum. There was no peritoneal fluid, but fortunately the bright light enabled him to distinguish between the peritoneum and the sac of the tumor, which were closely united by adhesions. The adhesions were broken down as far as the hand could reach. A trocar was next plunged into the tumor and about eighteen quarts, or about thirty-six pounds, of a dark, sanguineous fluid evacuted. The sac was enormously distended and very thin. After the most of the contents were drawn off, the opening made by the trocar was closed by Neláton's forceps. The sac was still adherent extensively laterally and posteriorly. These adhesions were destroyed as completely as possible, and yet it was impossible to draw the sac through the opening or to reach the deeper adhesions to the pelvis and flanks. Finally a free space was found in the right iliac fossa, the sac turned up and the pedicle found and secured. The greatest difficulty was found in separating the sac from the intestines, which were extensively adherent to the posterior surface of the sac, and could only be reached by turning the tumor upwards after division of the pedicle. A firm, broad adhesion to the stomach at the top of the cyst could not be separated, so a portion of the outer wall of the cyst, corresponding in size to the adhesion, was dissected from the tumor, and in that manner the entire tumor was finally removed. The pedicle was about two inches long and very slender, and came from the right side of the uterus. It was noticed in the specimen exhibited that the sac was almost entire, and that it was not smooth and glistening, but rough from the adhesions, and at the lower part there was an indurated mass about the size of a large orange.

The sac and mass together weighed but three pounds. There was a vast area of oozing surface from which the adhesions had been torn, yet very little blood was lost. The abdominal cavity was thoroughly sponged, a drainage tube introduced, and the wound closed by six silk sutures.

The length of the incision, after the removal of the tumor, was but two inches. The patient progressed favorably until the fourth day, when she was attacked with severe and persistent vomiting, rising temperature and pulse. The stercoraceous vomiting which ensued and the increasing exhaustion until her death, on the sixth day, pointed toward bowel obstruction.

The points to be noticed in this case are the great extent of the adhesions, the only clear space being in the right iliac fossa, the only way to reach them being to ligate and separate the pedicle and then turn the sac upwards; the small size of the pedicle for such a large tumor. It seemed to be approaching the conditions of a free ovarian tumor described by MR. DORAN, who explains their presence by atrophy and absorption of the pedicle taking place when there are very vascular adhesions of the tumor to other parts, through which nourishment is carried on. It was also noted that this patient went along all right for five days, then symptoms of intestinal obstruction set in. The question was raised, whether it is not the urgent duty of the surgeon in such a case to reopen the abdominal wound and search for the seat of the obstruction and remove it.

A CASE REQUIRING BATTEY'S OPERATION

Was next detailed by Professor Parkes. Miss M., age 17, single, an American, had suffered greatly from pain referred to right iliac fossa, increased during menstrual periods, for one year previous to placing herself under his care, January, 1885. She was already an invalid. Examination *per rectum et vaginam* disclosed retroversion of the uterus, accompanied with a dislocation of the right ovary into the Douglass *cul de sac*. Medical treatment having failed, Battey's operation for the removal of the prolapsed ovary was done June 2, 1885. Nothing unusual occurred during the operation, and the patient convalesced rapidly. The temperature never rose above 99°. The dressing placed upon the wound was the dry dressing consisting of iodoform, antiseptic gauze and a layer of absorbent cotton. This dressing was not removed until the seventh day, when the stitches were removed. The line of cicatrix was perfect throughout. There was no formation of pus. The blood around the stitch-holes was dry and scaly. The case was remarkable, principally, from the absolute freedom from any discomfort during the recovery from the operation. It was the first example of dry wounds Professor Parkes had ever seen. The patient was out in three weeks. The uterus was not fastened in its new position, and is slightly anteflexed, but not anteverted. The morbid specimen exhibited is the ovary much enlarged with two small parovarian cysts developed on it.

A CASE OF DOUBLE OVARIOTOMY

Closed the series of cases presented by Professor Parkes. Mrs. P., aet. 44, and had borne 5 children. She complained of a tumor growing for the last two years over the situation of the gall bladder. February 15, 1885, Professor Parkes made an exploratory operation, and after cutting into the growth, out popped a biliary calculus, about one inch long and bean-shaped. Nothing further was done, and the tumor began to disappear. However, during the manipulation of the abdominal walls he discovered two small tumors in the pelvis, in the region of the ovaries, which were supposed to be ovarian growths, but were not disturbed. But they rapidly grew in size, and were removed by laparotomy on June 16, 1885. The bowels had been moved two days previously. The abdominal incision disclosed the larger of the two tumors now shown, the larger filled the anterior inlet of the pelvis and was held down by slight adhesions. These were broken down and the mass removed. It was found to be the right ovary degenerated. The pedicle was extremely short and was with difficulty secured. It was tied, divided and the stump dropped. Likewise the left ovary was removed and found to be degenerated. The tumor being smaller and pedicle longer, no difficulty arose. Finally, in sponging, quite free bleeding was noticed from the right pedicle. It was again transfixed close to the uterus and all hemorrhage ceased.

It was noticed that the sigmoid flexure of the colon was distended with fæces, and this condition was remarked as unaccountable, from the fact that free catharsis had been obtained only two days previously. The patient did well for ten days after the operation, with the exception that no action of the bowels could be secured by medicines or enemas. A careful digital examination revealed, high up in the rectum, a stricture which could not admit the end of his index finger. The slight opening was surrounded on all sides by a thick, dense deposit of abnormal growth. The question of relief seemed to stand between forcible dilatation of the stricture and the establishment of an artificial anus. July 30, he dilated the stricture so as to admit three fingers in a cone shape. The tissue was very dense. It broke down with difficulty, and the induration extended up the bowel quite two inches. After this was done the scybalous mass was easily removed. By the next day the bowels had moved several times, freely, and tympany disappeared. As Professor Parkes had to leave the city July 5th, the patient was left in the care of Dr. R. G. Bogue, who subsequently told him the patient had died suddenly on July 10th, with symptoms of perforation of the bowels.

Two inquiries arise :

1st. Is it not well to examine the rectum thoroughly before operations, in all cases ?

2nd. Would the establishment of an artificial opening, as soon as the nature of the obstruction was determined, have given a better result in the case reported ?

Professor Parkes also exhibited A SPECIMEN OF EPITHELIOMA OF THE VULVA, which he had removed by Paquelin's cautery.

PROFESSOR ETHERIDGE, opened the discussion by asking how far we could go in removing portions of the intestine in cases of obstruction. He said that he had recently had a trying experience in case of ovarian tumor, in which, after the operation, the patient reacted well, but in forty-eight hours vomiting came on, which soon became stercoraceous and the patient died. On *post mortem* examination, the whole of the large and most of the small intestines were greatly distended with gas. A short piece of the small intestine, just where it entered the large one, was collapsed, and with great difficulty could any of the gas be forced through it. He asked the author of the paper if he would have had the courage to have removed this portion of the intestine.

DR. FRANKLIN H. MARTIN said he had been very much instructed and entertained by Professor Parkes' well detailed report. It had impressed him with three points or lessons that were dwelt upon in a recent article by A. VANDER VEER, M. D., of Albany, N. Y., in the July number of the *American Journal of Obstetrics*, citing "personal observations on the work of Lawson Tait," at his private hospital, in Birmingham, England. These three points, which to his mind explain, to a great extent, the wonderful results of that great operator, are :

1st. Cleanliness, pure air and sunlight.

2nd. Efficient good-looking nurses.

3rd. Close personal supervision, and attention to after-treatment.

In the first case Professor Parkes reported to-night that good light had saved him from going directly into the sac of the cyst in attempting to enter the peritoneum. This case also, he suggested, might have fared better had he been present to have given personal attention to the movement of the bowels, thus' possibly, removing the obstruction which was apparently present. In the third case, if personal attention had been given to the bowels sooner, the obstructions due to the stricture might have been removed and the ultimate result been more favorable. Here the nurse was at fault. Again, in the third case, if personal attention had been possible, the early operation for artificial anus, as Professor Parkes suggested, might have influenced favorably the ultimate result.

DR. MILLER thought that dilatation of the rectum was the only feasible method of relief in the case in which a malignant growth was formed in the lower bowel. An operation for an artificial anus would have been difficult under the circumstances.

PROFESSOR SARAH H. STEVENSON asked why not open the sigmoid flexure and remove the accumulation from it at the time of the operation?

PROFESSOR PARKES closed the discussion by saying he would remove a portion of the intestine if it presented the only way ot saving the patient's life. If in a few days after the operation symptoms of obstruction should come on, he would re-open the wound and seek for the seat of the trouble. He said that his theory of the best mode of procedure in these cases was gaining ground in the profession every day. DR. HAMILTON, of New York, lately reported a case of a gun-shot wound of the abdomen, in which there were eleven perforations of the small and four of the large intestine. These were sewed up and the patient recovered.

The cause of the constriction in the case reported by Professor Etheridge was probably due to an obstruction of the arterial supply to this part of the intestine, causing it to contract. Little good would come from an operation in this case. He thought an artificial anus might have been made in the second case referred to in his paper, though the bowel was distended. In these cases only a small opening was necessary. This might have prolonged the patient's life a short time. The cause of death was perforation of the bowels resulting from an ulcer incident to the dilatation of the rectum. He generally used saline cathartics to open the bowels. He would not have been justified in removing the accumulation from the sigmoid flexure at the time of operating, on account of the additional shock to the system it would have caused.

OBSERVATIONS ON THE CAUSE AND TREATMENT OF INFANTILE ECZEMA AND ALLIED ERUPTIONS, was the subject of a suggestive paper read by HENRY T. BYFORD, M. D. He said, in the winter of 1880 he had been called to attend Mrs. R. in her fifth confinement. Two of her other children were dead, and two more, apparently healthy at birth, had died in convulsions before they had completed one year of life. Both of the last two had suffered from scabby eruptions and had become somewhat emaciated, but had not been considered syphilitic. The child born at this time, seemingly healthy at first, soon broke out with what appeared to be an ordinary *eczema pustulosum*. This eruption occurred on the scalp and on different parts of the body. The child suffered from progressive emaciation, and at the age of three weeks it would easily have been mistaken for a case of struma, to be treated with cod-liver oil. The patient was quickly relieved by calomel in minute doses and mercurial inunctions.

The next case referred to was that of Augustus G., seen first in the summer of 1876. The child had pustular ezcema of the scalp; great nocturnal restlessness and suffered from progressive emaciation. There was no cause to suspect syphilis from the appearance of the child, but the father acknowledged to what seemed to be a perfectly cured attack of syphilis. The eczema rapidly disappeared, and the infant gained in flesh upon calomel powders and mercurial inunctions.

In contrast with these syphilitic cases, he next mentioned the case of E. H., a child one year of age, fat and well nourished, with no possibility of a syphilitic taint, but suffering from an eczema of the head, which spread over the body in patches. Prolonged local treatment had not improved it. The disease soon disappeared under the use of one-fifth grain powders of calomel, given twice a day, and an ointment composed of a dram of carbolic acid in an ounce of oxide of zinc ointment. The child had been over-fed and was put upon a restricted diet. There was no return of the disease.

The next case was one in which the eruption invaded the eyelids and caused great conjunctival sensitiveness. By the use of quarter-grain doses of calomel, given twice a day until a laxative effect was produced, then once a day, combined with the external application of carbolized oxide of zinc ointment and a borax eye-water, the condition of the patient was soon greatly improved.

He had succeeded in relieving many other severe cases of eczema by the use of calomel, and it made no difference on what part of the body the eruption occurred or what the condition of the patient was otherwise.

That eczema so frequently occurs in infancy, the large size and great activity of the liver in early life, and the striking action of calomel, had led the author to associate indigestion with infantile eczema as cause and effect. In syphilitic cases the alterative powders often produced an amelioration in the skin trouble sooner than they could through their direct action upon the blood-poison. In all cases it is important to regulate the diet.

He knew of no authority who had considered derangements of the liver, and its accompanying digestive disorders, as the chief cause of eruptions of the skin, or who had recommended calomel as the chief remedy for its cure.

As calomel produced such prompt relief, and as improvement of digestion usually followed rather than attended the action of the remedy, one is led to believe that the cure is brought about not merely by improving digestion but by a removal of waste, irritating, matter from the system, and from the great efficiency of mercury over other laxatives. He believed that the irritating materials are not only in the retained fecal matter but also in the blood—the products of imperfect digestion, assimilation and excretion.

He was led to the use of calomel in eczema from noting its good effect in cases due to syphilis, and as these patients improved so well under its use, he next tried it in cases where syphilis was only suspected, and then in all cases.

The usual dose was from one-quarter to one-eighth of a grain powder, given twice a day, the dose to be reduced if too great irritation of the bowels was produced. In cases of children over two and a half years of age, in order to avoid salivation, he usually gave purgative doses of calomel every six or eight days and trusted to diet and other remedies in the meantime.

DR. E. J. DOERING said he had not used calomel in eczema, but had had good results from the use of corrosive sublimate. He intends to use calomel, after this, in such cases, and he thought nothing better could be found to use in chronic cases.

DR. J. ZEISLER remarked that he was very much interested in this paper and, especially, to hear that calomel was useful in eczematous skin diseases. He had always held the view advocated by HEBRA, that eczema was caused by external causes and required external applications for its cure. Calomel might be of use in cases due to syphilis. He would not use blue ointment on young children. He asked whether it would not be best to use some external application in cases where there were thick masses of crusts covering the diseased part, and also whether the author of the paper had found salicylic acid useful in this disease.

DR. C. W. PURDY thought retained excreta a cause of eczema, and called attention to the close relation existing between the intestinal tract and the skin, the condition of the one affecting the other. He had seen cases cured by the use of calomel and would favor this treatment.

DR. J. A. ROBISON said that Dr. Byford had found mercury beneficial in syphilitic and non-syphilitic cases, probably because given in small doses it was a general tonic, as iron or cod-liver oil, increasing the number of red blood corpuscles and enriching the blood. Thus it counteracted any dyscrasia present.

DR. BYFORD concluded by saying that Hebra was doubtless right in saying that eczema is caused often by external causes, but in some cases it was clear to him that the disease is due to causes arising within the system itself. In later years Hebra had modified his ideas of this disease somewhat. Although eczema could be cured by external applications, when so treated it is very apt to return. It is not so when removed by the use of calomel. External medication would often hasten the cure. He did not use salicylic acid. He believed that calomel in these cases acted as an agent causing eliminations as well as being a tonic.

